# Patients presenting with acute poisoning to an outpatient emergency clinic: a one-year observational study in Oslo, Norway

**DOI:** 10.1186/s12873-015-0045-2

**Published:** 2015-08-13

**Authors:** Odd Martin Vallersnes, Dag Jacobsen, Øivind Ekeberg, Mette Brekke

**Affiliations:** Department of General Practice, University of Oslo, Oslo, Norway; Oslo Accident and Emergency Outpatient Clinic, Department of Emergency General Practice, City of Oslo Health Agency, Oslo, Norway; Department of Acute Medicine, Oslo University Hospital, Oslo, Norway; Division of Mental Health and Addiction, Oslo University Hospital, Oslo, Norway; Department of Behavioural Sciences in Medicine, University of Oslo, Oslo, Norway

## Abstract

**Background:**

In Oslo, the majority of patients with acute poisoning are treated in primary care, at an emergency outpatient clinic with limited diagnostic and treatment resources. We describe the poisonings currently seen in this setting. We compare our findings with previous studies, with special concern for the appearance of new toxic agents, and changes in overall numbers and patterns of poisoning.

**Methods:**

Observational study. Patients above the age of 12 years presenting at Oslo Accident and Emergency Outpatient Clinic (Oslo Legevakt) with acute poisoning were included consecutively from October 2011 through September 2012. Physicians and nurses registered data on preset forms. Main outcome measures were toxic agents, age, sex, intention, referral and time of presentation.

**Results:**

There were 2923 episodes of acute poisoning in 2261 patients. Median age of the patients was 32 years, and 1430 (63 %) were males. The most frequent toxic agents were ethanol in 1684 (58 %) episodes, heroin in 542 (19 %), benzodiazepines in 521 (18 %), amphetamine in 275 (9 %), fire smoke in 192 (7 %), gamma-hydroxybutyrate (GHB) in 144 (5 %), and cannabis in 143 (5 %). In 904 (31 %) poisonings there were more than one toxic agent. In 493 episodes (17 %), the patient was hospitalised, and in 60 episodes (2 %) admitted to a psychiatric ward. Most poisonings, 2328 (80 %), were accidental overdoses with substances of abuse, 276 (9 %) were suicide attempts, and 312 (11 %) were accidents. Among ethanol poisonings in patients above the age of 26 years, 685/934 (73 %) were in males, and 339/934 (36 %) presented during weekends. However, among ethanol poisonings in patients under the age of 26 years, 221/451 (49 %) were in females, and 297/451 (66 %) presented during weekends.

**Conclusions:**

The poisonings treated in this primary care setting were mostly due to accidental overdoses with ethanol or other substances of abuse. There is a disconcerting weekend drinking pattern among adolescents and young adults, with young females presenting as often as young males with ethanol poisoning.

## Background

Acute poisoning constitutes a major health problem. It causes frequent presentations to emergency services, and is mainly due to suicidal behaviour, substance use disorders, or accidents. Irrespective of intention, the long-term mortality is high for this patient group [1]. The pattern of poisoning varies between areas and changes over time. New drugs appear [2, 3], poisoning patterns for familiar substances change [4, 5], there are local endemics [6] and epidemics [7, 8]. To keep track of trends and changes, updated studies are needed.

Most patients with acute poisoning are treated as outpatients in hospital emergency departments. In Oslo, however, the majority of poisoned patients are treated in primary care, at the Oslo Accident and Emergency Outpatient Clinic (OAEOC, Oslo Legevakt). Previous studies in 2003 and 2008 have shown that treatment of acute poisoning at the OAEOC is safe, considering the low mortality during treatment and immediately after discharge [9, 10].

The prognosis for patients treated for acute poisoning is serious, whether the poisoning was a suicide attempt or associated with substance abuse [1, 11, 12]. The repetition rate is high; one in three patients present with acute poisoning again within the first year [13]. Follow-up is usually initiated after suicide attempt, but less frequently for patients with substance abuse [14–17]. This is also the case at the OAEOC [9]. In our study from 2008 we assessed follow-up initiated from the OAEOC [9], but not whether the patients actually presented at the institution or clinic they were referred to. In the present study, carried out in 2012, this will be addressed. This article presents the epidemiological data from the 2012 study. The follow-up data will be presented later.

The number of poisonings treated at the OAEOC more than doubled from 2003 to 2008, in contrast to the stable incidence of poisoning seen at Oslo hospitals in the same time period [9, 10]. This trend needs to be tracked. Furthermore, as the majority of poisonings with substances of abuse in Oslo are treated at the OAEOC [9, 15], it is well suited as a sentinel post for assessing changes in their epidemiology.

### Objective

The objective was to describe the current poisoning pattern seen at the OAEOC, with special concern for the appearance of new toxic agents and changes in overall numbers, patterns of poisoning, intention and referral. The study was designed similarly to the studies in 2003 and 2008 [9, 10], in order to compare results.

## Methods

### Design

The study was an observational cross-sectional study, part of a larger prospective study of treatment of acute poisoning at the OAEOC.

### Setting

The Norwegian emergency care system has two levels; hospitals and primary care emergency services. Local primary care emergency services are provided by regular general practitioners during office hours, and by general practice out-of-hours services in local casualty clinics during nights and weekends. Some casualty clinics are in service at all hours. Patients cannot present directly to hospital emergency departments, but have to be assessed in primary care or by the ambulance service first.

The OAEOC is the main casualty clinic in Oslo, serving the entire city at all hours. It has about 200 000 consultations a year, equally split between departments for accidents and for emergency general practice. It also contains emergency social services and a psychiatric emergency outpatient clinic. There are facilities for observation of poisoned patients for up to four hours. However, diagnostic tools and treatment options are limited, making the management of poisoned patients less costly than in hospitals. Other casualty clinics in Oslo do not treat patients with acute poisoning to any significant extent. About one in ten patients presenting to the OAEOC are referred to hospital. Oslo is the capital of Norway and as of January 1^st^ 2012 had 613 285 inhabitants, of whom 502 336 were above the age of 12.

### Inclusion

All patients above the age of 12 treated at the OAEOC with a main diagnosis of acute poisoning were included. Patients with other main diagnoses, but a co-diagnosis of acute poisoning, were included if the poisoning necessitated treatment. The study period was set to one year to encompass seasonal variations. Patients were included consecutively from October 1^st^ 2011 to September 30^th^ 2012, by the physician treating them. Patient lists in the electronic medical records were regularly and systematically searched during the study period to ensure the inclusion of all eligible patients. Any eligible patients not included while treated, were included when found in these searches.

### Data collection

The physician and nurse treating the patient registered data on preset forms. We reviewed the registration forms, and any missing information was collected, if available, from the local electronic medical records. Data for patients included from the patient list searches was collected from the local electronic medical records.

### Outcome measures

The outcome measures were toxic agents, age, sex, intention, referral, and time and mode of presentation.

The physician treating the patient made diagnoses of toxic agents based on all information available at the time, including statements from the patient and companions, clinical examination, and information from the police or ambulance service. No toxicological screening tests are done at the OAEOC, except for ethanol breath analyser test. In poisonings with more than one toxic agent, the main agent was defined as the one considered most toxic in the doses taken.

The physician also categorised intention. Poisonings with substances of abuse (including ethanol, opioids, benzodiazepines, Z-hypnotics, gamma-hydroxybutyrate (GHB), cannabis, cocaine, amphetamine and related substances, hallucinogens, other novel psychoactive substances, and other substances with abuse potential) taken for intoxication or recreational purposes were classified as accidental overdoses with substances of abuse (AOSAs). Poisonings with any degree of suicidal intention were classified as suicide attempts. Poisonings with no intention of self-harm or intoxication were classified as accidents.

Time of presentation was categorised as weekend or weekday. Weekend was defined as presentation between Saturday 00:00 and Sunday 23:59.

### Ethics

The study was performed in accordance with the Helsinki declaration. It was approved by the Regional Committee South East for Medical and Health Research Ethics (REK nr 2010/1129–1) and by the Privacy Protection Ombudsman at Oslo University Hospital.

### Statistics

Analyses were done in IBM SPSS version 21 (IBM Corp.). Pearson’s chi-square test or Fisher’s exact test (for expected cell values five or less) were used to compare frequencies. Mann-Whitney *U*-test was used in age comparisons.

## Results

There were 3139 episodes of acute poisoning during one year. In 216 (7 %) episodes, the patient declined participation, leaving 2923 cases of acute poisoning in 2261 patients included in the study. There were 1430 (63 %, *p* < 0.001) male patients, and 1919 (66 %, *p* < 0.001) of the poisoning episodes occurred in males. Median age was 32 years, 35 years for males and 28 years for females (Table 1). There was no significant difference in age (*p* = 0.81) or sex (*p* = 0.75) between the included patients and those who declined.

**Table 1:** Demographic data for 2261 patients treated for acute poisoning at Oslo Accident and Emergency Outpatient Clinic during one year

	Males *n* (%)	Females *n* (%)	Total
Certain identity^a^	1301 (91)	794 (96)**	2095 (93)
Uncertain identity^b^	104 (7)	30 (4)**	134 (6)
Unknown identity	25 (2)	7 (1)*	32 (1)
Episodes:			
1	1213 (85)	740 (89)**	1953 (86)
2–5	201 (14)	87 (10)**	288 (13)
6–25	16 (1)	4 (<1)*	20 (1)
Age:			
12–15	13 (1)	15 (2)	28 (1)
16–25	374 (26)	353 (42)	727 (32)
26–35	345 (24)	157 (19)**	502 (22)
36–45	286 (20)	121 (15)**	407 (18)
46–55	224 (16)	97 (12)**	321 (14)
56–65	103 (7)	46 (6)**	149 (7)
66–75	46 (3)	17 (2)**	63 (3)
>75	15 (1)	20 (2)	35 (2)
Unknown	24 (2)	5 (1)**	29 (1)
Total individuals	1430 (100)	831 (100)**	2261 (100)

^a^Persons with National Registry personal identity number

^b^Persons stating their identity, but without National Registry personal identity number

P-values are given for comparisons of numbers of patients between sexes

*Significant difference between sexes, *p* < 0.05

**Significant difference between sexes, *p* < 0.001

The most frequent toxic agents were ethanol in 1684 (58 %) episodes, heroin in 542 (19 %), benzodiazepines in 521 (18 %), and amphetamine in 275 (9 %) (Table 2).

**Table 2:** Toxic agents in 2923 acute poisonings treated at Oslo Accident and Emergency Outpatient Clinic during one year

	All episodes	Episodes as main agent^a^			Episodes as co-agent			Episodes with suicidal intention^b^		
	*n* (%)	Total *n* (%)	Males *n* (%)	Females *n* (%)	Total *n* (%)	Males *n* (%)	Females n (%)	Total *n* (%)	Males* n* (%)	Females *n* (%)
Ethanol	1684 (58)	1400 (48)	928 (48)	472 (47)**	284 (23)	174 (22)	110 (26)**	14 (5)	5 (6)	9 (5)
Heroin	542 (19)	490 (17)	391 (20)	99 (10)**	52 (4)	42 (5)	10 (2)**	13 (5)	10 (11)	3 (2)
Benzodiazepines	521 (18)	201 (7)	115 (6)	86 (9)*	320 (26)	231 (29)	89 (21)**	94 (34)	35 (39)	59 (32)*
Amphetamine	275 (9)	108 (4)	79 (4)	29 (3)**	167 (14)	131 (16)	36 (8)**	3 (1)	1 (1)	2 (1)
Fire smoke	192 (7)	191 (7)	113 (6)	78 (8)*	1 (<1)	0 (0)	1 (<1)	0 (0)	0 (0)	0 (0)
GHB	144 (5)	115 (4)	90 (5)	25 (2)**	29 (2)	21 (3)	8 (2)*	1 (<1)	1 (1)	0 (0)
Cannabis	143 (5)	30 (1)	16 (1)	14 (1)	113 (9)	79 (10)	34 (8)**	0 (0)	0 (0)	0 (0)
Paracetamol	70 (2)	48 (2)	10 (1)	38 (4)**	22 (2)	4 (1)	18 (4)*	42 (15)	8 (9)	34 (18)**
Methadone	54 (2)	34 (1)	23 (1)	11 (1)*	20 (2)	14 (2)	6 (1)	1 (<1)	0 (0)	1 (1)
Cocaine	53 (2)	16 (1)	12 (1)	4 (<1)*	37 (3)	26 (3)	11 (3)*	1 (<1)	1 (1)	0 (0)
Antipsychotics	46 (2)	30 (1)	4 (<1)	26 (3)**	16 (1)	3 (<1)	13 (3)*	27 (10)	4 (4)	23 (12)**
Antidepressives	39 (1)	24 (1)	6 (<1)	18 (2)*	15 (1)	4 (1)	11 (3)	23 (8)	5 (6)	18 (10)*
Buprenorphine	38 (1)	22 (1)	16 (1)	6 (1)*	16 (1)	12 (2)	4 (1)*	1 (<1)	0 (0)	1 (1)
NSAIDs	31 (1)	14 (<1)	3 (<1)	11 (1)*	17 (1)	7 (1)	10 (2)	14 (5)	3 (3)	11 (6)*
Ecstasy	18 (1)	8 (<1)	7 (<1)	1 (<1)	10 (1)	6 (1)	4 (1)	0 (0)	0 (0)	0 (0)
Other opioids	64 (2)	27 (1)	19 (1)	8 (1)*	37 (3)	16 (2)	21 (5)	7 (3)	5 (6)	2 (1)
Other substances of abuse	71 (2)	48 (2)	34 (2)	14 (1)*	23 (2)	15 (2)	8 (2)	1 (<1)	1 (1)	0 (0)
Other pharmaceuticals	89 (3)	52 (2)	17 (1)	35 (3)*	37 (3)	8 (1)	29 (7)*	31 (11)	10 (11)	21 (11)*
Other^c^	74 (3)	65 (2)	36 (2)	29 (3)	9 (1)	4 (1)	5 (1)	3 (1)	1 (1)	2 (1)
Total	2923 (100)^d^	2923 (100)	1919 (100)	1004 (100)**	1226 (100)	797 (100)	429 (100)**	276 (100)	90 (100)	186 (100)**

^a^The main agent was defined as the one most toxic in the doses taken

^b^Main agents only

^c^Including seven patients initially diagnosed with acute poisoning, but during observation found to have other causes for their condition

^d^Percentages add up to more than 100 as 904 poisonings had more than one agent

P-values are given for comparisons between sexes of absolute numbers of episodes within group per agent

*Significant difference between sexes, *p* < 0.05

**Significant difference between sexes, *p* < 0.001

GHB: Gamma-hydroxybutyrate

NSAIDs: Non-steroid anti-inflammatory drugs

In 904 (31 %) episodes there were more than one toxic agent. Among these, 647 had one co-agent, 194 had two co-agents, 63 had from three to five co-agents, and 599/904 (66 %, *p* < 0.001) were in males. Most common were combinations of opioids with benzodiazepines or central stimulants (Table 3).

**Table 3:** Combinations of toxic agents in 2923 acute poisonings treated at Oslo Accident and Emergency Outpatient Clinic during one year

			Co-agent						
Main agent	Main agent^a^ total* n* (%)	Episodes with co-agents *n* (%)^b^	Benzodiazepines *n* (%)	Ethanol n (%)	Central stimulants *n* (%)	Opioids n (%)	Cannabis *n* (%)	Pharmaceuticals *n* (%)	Other n (%)
Ethanol	1400 (48)	197 (14)	74 (23)	–	43 (20)	20 (16)	57 (50)	14 (13)	24 (39)
Opioids	573 (20)	299 (52)	187 (58)	61 (21)	98 (46)	34 (27)^c^	19 (17)	10 (9)	15 (24)
Benzodiazepines	201 (7)	119 (59)	–	72 (25)	26 (12)	29 (23)	15 (13)	20 (19)	4 (6)
Fire smoke	191 (7)	23 (12)	–	23 (8)	–	–	–	–	1 (2)
Pharmaceuticals	168 (6)	89 (53)	21 (7)	39 (14)	4 (2)	14 (11)	2 (2)	59 (55)^c^	4 (6)
Central stimulants	132 (5)	72 (55)	20 (6)	29 (10)	14 (7)^c^	19 (15)	10 (9)	3 (3)	12 (19)
GHB	115 (4)	63 (55)	16 (5)	32 (11)	25 (12)	4 (3)	4 (4)	1 (1)	1 (2)
Other	143 (5)	42 (29)	2 (1)	28 (10)	4 (2)	5 (4)	6 (5)	1 (1)	1 (2)^c^
Total	2923 (100)	904 (31)	320 (100)	284 (100)	214 (100)	125 (100)	113 (100)	108 (100)	62 (100)

^a^The main agent was defined as the one most toxic in the doses taken

^b^Percentage of poisoning episodes for given main agent with multiple agents

^c^The combinations of opioids with opioids, pharmaceuticals with pharmaceuticals, and central stimulants with central stimulants, are combinations of different categories of these agents, e.g. heroin with methadone

GHB: Gamma-hydroxybutyrate

**Fig. 1:**
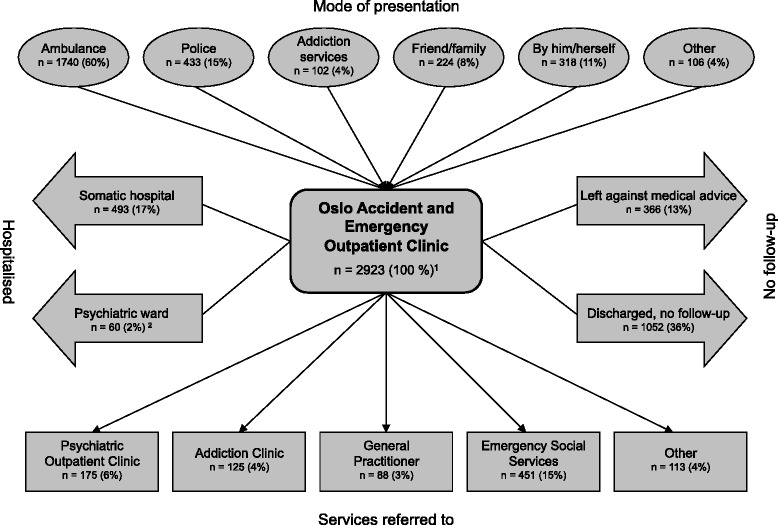
Patient flow in 2923 cases of acute poisoning presenting to Oslo Accident and Emergency Outpatient Clinic (OAEOC) during one year. (^1^Excludes 216 cases where the patient declined participation. ^2^Includes 28 patients admitted for compulsory observation)

Patient flow is presented in Fig. 1. One in five patients were hospitalised. No patients died at the OAEOC.

**Fig. 2:**
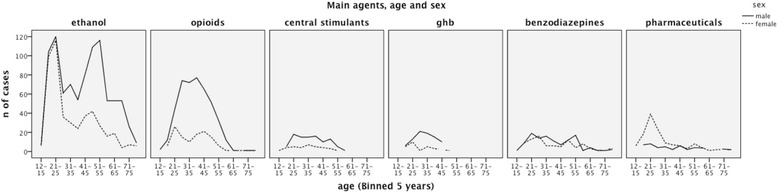
Age, sex and main toxic agents. Age and sex distribution for groups of main agents among poisonings treated at Oslo Accident and Emergency Outpatient Clinic during one year. For ethanol poisoning, the peaks in the young age groups were due to many patients with one poisoning episode. The peak among older males was due to a group of patients with repeated poisonings

Poisonings with illegal substances of abuse were more common in males, and pharmaceuticals in young females (Fig. 2). While ethanol poisonings overall were more common in males, among the 451 ethanol poisonings in patients under the age of 26 years, 221 (49 %, *p* = 0.67) were in females. Of the 37 patients with five or more poisoning episodes, 20 (54 %) were males aged from 36 to 60. They mainly presented with ethanol or heroin poisonings.

Most poisonings, 2328 (80 %), were AOSAs, 276 (9 %) were suicide attempts, and 312 (11 %) were accidents. AOSAs were more frequent among males, 1652/2328 (71 %, *p* < 0.001). Suicide attempts were more frequent among females, 186/276 (67 %, *p* < 0.001). Accidents were more evenly distributed, with 171/312 (55 %, *p* = 0.09) occurring among males. Among the suicide attempters 24/276 (9 %) were admitted to psychiatric wards (including four patients admitted for compulsory observation), 123/276 (45 %) admitted to somatic hospitals, 79/276 (29 %) referred to psychiatric outpatient clinics, and 25/276 (9 %) to other follow-up, while 6/276 (2 %) were discharged with no follow-up and 19/276 (7 %) left against medical advice (Fig. 1). Benzodiazepines and paracetamol were the most common main agents taken in suicide attempts (Table 2). Most of the accidents, 191/312 (61 %), were exposures to fire smoke, requiring hospitalisation in 27/191 (14 %) cases.

Young patients with ethanol poisoning more often presented during weekends than on weekdays; 297/451 (66 %, *p* < 0.001) of those under the age of 26 years, 105/198 (53 %, *p* < 0.001) of those aged 26 to 35 years, and 76/196 (39 %, *p* = 0.002) of those aged 36 to 45 years, presented on Saturdays or Sundays. This weekend presentation trend for ethanol poisoning was not seen among patients above the age of 45. Ethanol poisonings in weekends were more common in males, but among patients under the age of 26, 143/297 (48 %, *p* = 0.52) were in females. GHB poisonings, 50/115 (43 %, *p* < 0.001), were also more frequent during weekends.

## Discussion

### Summary of main results

The majority of the 2923 poisonings were AOSAs, and the most frequent toxic agents were ethanol, heroin, benzodiazepines, and amphetamine. One in five patients were hospitalised. Half of the ethanol poisonings in patients under the age of 26 were in females. Most young patients with ethanol poisoning presented during weekends.

### Strengths and limitations

Patients were registered consecutively during one year to ensure that all poisonings were included, and to encompass seasonal variations. Though some patients declined to participate, the study gives a nearly complete picture of the poisonings seen at the OAEOC. It does not, however, give the full picture of poisoning in Oslo, mainly because the most severely poisoned patients are not treated at the OAEOC. There are approximately 120 deaths from poisoning in Oslo annually, 80–90 % occur outside the health services and are declared dead on site [15, 18]. Some 700 poisonings per year bypass the OAEOC on their way to Oslo hospitals, most of them poisonings with pharmaceuticals triaged by the ambulance service [10, 15]. In 2003, 750 patients were discharged on site by the ambulance service, mainly after treatment for opioid poisoning [10]. However, most poisonings with substances of abuse in Oslo are treated at the OAEOC [9, 15]. Thus, this study should be a good indicator of trends and changes in this category of poisonings in Oslo.

No toxicological screening tests are done at the OAEOC, except for ethanol breath analyser test. No confirmative tests were done in this study, rendering the diagnosis of toxic agents uncertain. This is a major limitation. However, the study mirrors the actual clinical situation in emergency settings such as the OAEOC. There may also be inter-rater differences in the diagnostic assessments, as many physicians participated.

Some co-categorisations make the results less precise. Both amphetamine and methamphetamine are probably present in the amphetamine category. Users in Oslo rarely distinguish between them, but Norwegian police has seized more methamphetamine than amphetamine since 2003 [19]. In 2008 Z-hypnotics made up 15 % of the sedatives [9]. Developments, however, cannot be traced, as Z-hypnotics were categorised as benzodiazepines in the present study. From comments written on the registration forms it is evident that poisonings with synthetic cannabinoids first appeared at the OAEOC in the fall of 2011. However, no separate category had been made on the registration forms, and they were categorised as cannabis.

### Trends and changes

The number of poisonings treated at the OAEOC has increased by 24 % since 2008 [9]. In comparison, the population of Oslo has grown by 8 %. Thus, the increase observed from 2003 to 2008 [9] has continued. GHB poisonings more than doubled (148 % increase), and there were substantial increases in ethanol (36 %), amphetamine (40 %) and cocaine (66 %) poisonings. In contrast, there was a decrease in fire smoke exposures. The proportions of ambulance referrals, hospital admissions and suicide attempts were largely unchanged since 2008 [9].

Total sales of alcohol in Norway has been stable since 2007, and total consumption among adolescents and young adults had been decreasing from 2003 to 2008 [19]. Still, the number of acute ethanol poisonings at the OAEOC has increased, many of them, as elsewhere [20, 21], in young patients in weekends. Studies from different European cities also report an increase in the number of ethanol poisonings over the last decade, especially among young adults and adolescents [4, 21–23]. It is possible that a segment of the younger population drink larger quantities than before, as some studies have shown [5, 23, 24]. If so, this is a disconcerting trend, as binge drinking carries the risk of serious consequences [25, 26].

Half of the young patients with ethanol poisoning were female. Though most studies report a majority of males [4, 5, 20, 21], similar findings have been reported from Slovakia [23], and a Swiss study showed an increasing number of young females with ethanol poisoning [22]. About 80 % of Norwegians under the age of 20 report having drunk alcohol. The proportion has been slightly larger for females than males during the last decades [19]. However, young females presenting with ethanol poisoning as often as young males, has not been reported in Norway before.

The increase in GHB poisoning is probably a reflection of more widespread use in Norway, consistent with the increase in police seizures [19].

Poisonings with novel central stimulant drugs were hardly seen. This contrasts with findings from the UK [2]. Ecstasy poisonings were few, similar to in the UK [2], and consistent with decreasing seizures by Norwegian police [19]. Poisoning with these substances may have been misclassified as amphetamine or cocaine, both increased compared to 2008 [9]. Amphetamine still dominated, as has been usual in Norway [27].

The widespread combination of opioids with benzodiazepines and central stimulants is worrying [28, 29]. Most fatal poisonings in Oslo are due to opioids, usually combined with other agents [15, 18, 30].

In 2008, 48 % of the patients were discharged without follow-up [9]. This proportion was reduced to 36 % in this study, mainly because many patients were enrolled in a new follow-up program for the young run by the Emergency Social Services. A major incitement for creating this program was the large proportion of patients discharged without follow-up shown in the 2008 study. The emergency setting is an opportunity for interventions to reduce alcohol and drug use and abuse, and such interventions should be encouraged [31, 32].

Only two percent of patients with suicidal intention were discharged without follow-up, a reassuring finding, as these patients have a higher risk of future suicide [1]. Compared to 2008, patients were referred to psychiatric outpatient treatment rather than hospitalised, after suicide attempts [9]. More detailed data on the follow-up initiated from the OAEOC, and whether the patients actually presented at the institution they were referred to, will be presented in separate articles.

### Acute poisoning in primary care

This study shows the wide range of poisonings treated in a primary care setting. Four fifths of the patients with acute poisoning brought to the OAEOC were treated locally and discharged without transfer to hospital, as in the previous studies [9, 10], and no patients died at the OAEOC. As long as the patients in need of hospital management are identified and transferred, it seems sensible to treat poisoned patients in primary care outpatient emergency clinics.

## Conclusions

A large and increasing number of acute poisonings were treated at the OAEOC, mostly accidental overdoses with ethanol or other substances of abuse. There is a disconcerting weekend drinking pattern among adolescents and young adults, with young females presenting as often as young males with ethanol poisoning.

## Abbreviations

AOSA: Accidental overdose with substances of abuse
GHB: Gamma-hydroxybutyrate
NSAIDs: Non-steroid anti-inflammatory drugs
OAEOC: Oslo Accident and Emergency Outpatient Clinic
UK: United Kingdom of Great Britain and Northern Ireland
Z-hypnotics: Zolpidem and zopiclone
